# Institutional Organisation in America

**Published:** 1914-08-15

**Authors:** 


### INSTITUTIONAL ORGANISATION IN AMERICA.*

### IV. Conundrums About Hospital Efficiency.

We have already, in a previous article, dealt with the organisation of one department of the Massachusetts General Hospital, Boston. Another institution in this same city, the Boston Dispensary, has compiled some conundrums on the subject of hospital efficiency. In so far as they illustrate the tendencies of the moment in the American institutional world, they are worthy of quotation.

The first one is, When the mother needs an operation and the father needs a job, is it fair to the children for the hospital to provide a surgeon without a social service department? The next one reads, Remembering that a surgeon's grandfather laughed at asepsis, are you surprised when his grandson smiles at social service?

The trend of these two questions is sufficiently obvious not to require an answer in the minds of those who have formulated them. The first question offends the elementary canons of logic in not defining the term hospital, and both of them are *argumenta ad hominem* rather than to pure reason. The third question will, however, command wider agreement. It asks, When is a dispensary for the poor a poor dispensary? and the reply is given, When it hasn't enough money to provide efficient treatment for its patients, nor enough imagination to prepare a programme which will convince the public of its needs.

The fourth question is, Why is a hospital without a convalescent home like a laundry which washes the clothes, but does not dry and press them ready for wear? The fifth one asks, Is it worth while to provide a brace for the support of a rachitic child without providing enough income for the support of the family? And the last one is, After tabulating the proportion of patients who paid only one visit to your clinic, would you be willing to write a paper on the efficacy of absent treatment?

It will be observed that these questions are of the rhetorical order. They suggest the answers which seem to their composers to be the correct ones, and there is nothing in them of any very special originality or profundity. Still, they do show that a very wholesome spirit of self-inquiry is abroad in the American institutional world, as indeed the preceding articles of this series have proved abundantly already.

The phrase hospital efficiency is one which is capable of various interpretations. Some committees in this country are inclined to attach too much importance to statistical returns of the average length of stay in hospital, or of the mortality after operation, or of some similar factor. To judge by certain comments in the (American) *Medical Review of Reviews* this spirit also occasionally dominates conceptions of hospital efficiency in the United States. As a matter of fact, the desire to reduce to the lowest possible number of days the average length of stay of each patient in a hospital is frequently inimical to true efficiency. Committees which press their medical staff on points such as this may do much harm, for their action may result in the discharge of patients who are only semi-convalescent, and by no means fit to return to their homes. "The restoration of a patient to society in proper condition to enable him to take up his duties and responsibilities is one test of the efficiency of present-day hospital methods. By this test most hospitals would fail to be classified as satisfactory social agents." If the Socratic method of interrogation which is just now popular in the United States elicits and establishes truths of this nature, it will prove of far-reaching value to true hospital efficiency. The institutional spirit is clearly in active operation in Boston, and must surely be leading to an ever higher standard of service to society and the State.

* Articles I., II., and III. appeared on July 11, 18, and August 1 respectively.

